# Supplementary material for: Effects of Low and High Maternal Protein Intake on Fetal Skeletal Muscle miRNAome in Sheep
Source: Animals (Basel). 2024 May 28;14(11):1594. doi: 10.3390/ani14111594 (PMC11171157; doi:10.3390/ani14111594)
Supplement: Supplementary file 1 [file animals-14-01594-s001.zip › Tables S1,S4-S7.pdf]

Table S1. Ewe and fetus specifications.

**Parameters**	**Standard Protein**	**High protein**	**Low protein**	**SEM**	***p*-value**
Number	4	4	4		
Breed	Akkaraman	Akkaraman	Akkaraman	-	-
Age	2 years	2 years	2 years	-	-
Live Weight at the beginning (kg)	58.40	57.00	57.00	2.004	0.954
30. day live weight (kg)	58.22	57.94	57.96	2.089	0.998
105. day live weight (kg)	75.50	74.00	62.50	4.631	0.568
Fetal weight (kg)	1.09	1.11	1.13	0.22	0.587

Table S4. GO terms of 32 genes targeted by multiple miRNAs with ConsensusPathDB (category level 4) (adjusted $p<0.01$).

**Category**	**Gene ontology term**		**Set size**	**Candidates contained**	***p*-value**	***q*-value**
**Biological process**	GO:0048247	lymphocyte chemotaxis	65	3 (4.6%)	9.74e-05	0.00792
	GO:0002548	monocyte chemotaxis	71	3 (4.2%)	0.000127	0.00792
	GO:0051171	regulation of nitrogen compound metabolic process	6033	17 (0.3%)	0.000574	0.0199
	GO:0071621	granulocyte chemotaxis	124	3 (2.4%)	0.000655	0.0199
	GO:0080090	regulation of primary metabolic process	6227	17 (0.3%)	0.000863	0.0199
	GO:0031323	regulation of cellular metabolic process	6297	17 (0.3%)	0.000996	0.0199
	GO:0097530	granulocyte migration	149	3 (2.0%)	0.00111	0.0199
	GO:0006796	phosphate-containing compound metabolic process	3342	11 (0.3%)	0.0032	0.0419
	GO:0044271	cellular nitrogen compound biosynthetic process	5019	14 (0.3%)	0.0032	0.0419
	GO:0009059	macromolecule biosynthetic process	5040	14 (0.3%)	0.00335	0.0419
	GO:0060255	regulation of macromolecule metabolic process	6339	16 (0.3%)	0.0037	0.042
	GO:0044267	cellular protein metabolic process	5282	14 (0.3%)	0.00531	0.0553
	GO:0034976	response to endoplasmic reticulum stress	298	3 (1.0%)	0.00779	0.0602
	GO:0034654	nucleobase-containing compound biosynthetic process	4307	12 (0.3%)	0.00786	0.0602
	GO:0060326	cell chemotaxis	305	3 (1.0%)	0.00837	0.0602
	GO:0048638	regulation of developmental growth	306	3 (1.0%)	0.00845	0.0602
	GO:0018130	heterocycle biosynthetic process	4372	12 (0.3%)	0.00889	0.0602
	GO:0019438	aromatic compound biosynthetic process	4383	12 (0.3%)	0.00908	0.0602
	GO:0034645	cellular macromolecule biosynthetic process	4976	13 (0.3%)	0.00916	0.0602
**Molecular function**	GO:0005125	cytokine activity	237	4 (1.7%)	0.000299	0.00459
	GO:0005126	cytokine receptor binding	273	4 (1.5%)	0.00051	0.00459
	GO:0019887	protein kinase regulator activity	190	3 (1.6%)	0.00224	0.0134
	GO:0048018	receptor ligand activity	493	4 (0.8%)	0.00445	0.0164
	GO:0019210	kinase inhibitor activity	73	2 (2.7%)	0.00456	0.0134
	GO:0001664	G protein-coupled receptor binding	294	3 (1.0%)	0.00757	0.0227

Table S5. Pathway analysis of 32 genes targeted by multiple miRNAs with ConsensusPathDB (adjusted *p*<0.01).

Pathway name	Set size	Candidates contained	*p*-value	*q*-value	Pathway source
Chemokine receptors bind chemokines	62	3 (4.8%)	0.000117	0.00397	Reactome
COVID-19 adverse outcome pathway	15	2 (13.3%)	0.000241	0.00397	Wikipathways
Rheumatoid arthritis - Homo sapiens (human)	93	3 (3.3%)	0.000378	0.00397	KEGG
Viral protein interaction with cytokine and cytokine receptor - Homo sapiens (human)	100	3 (3.0%)	0.000483	0.00397	KEGG
Chagas disease - Homo sapiens (human)	102	3 (2.9%)	0.000512	0.00397	KEGG
Toll-like receptor signaling pathway - Homo sapiens (human)	104	3 (2.9%)	0.000542	0.00397	KEGG
IL-7 signaling pathway	25	2 (8.0%)	0.000683	0.00429	Wikipathways
Cytokine-cytokine receptor interaction - Homo sapiens (human)	295	4 (1.4%)	0.00102	0.00558	KEGG
Cyclin D associated events in G1	44	2 (4.5%)	0.00212	0.00931	Reactome
G1 Phase	44	2 (4.5%)	0.00212	0.00931	Reactome
Chemokine signaling pathway - Homo sapiens (human)	192	3 (1.6%)	0.00316	0.0126	KEGG
Peptide ligand-binding receptors	207	3 (1.4%)	0.00391	0.0143	Reactome
Lipid and atherosclerosis - Homo sapiens (human)	215	3 (1.4%)	0.00435	0.0146	KEGG
Human cytomegalovirus infection - Homo sapiens (human)	225	3 (1.3%)	0.00493	0.0146	KEGG
DNA damage response	68	2 (2.9%)	0.00498	0.0146	Wikipathways
E2F transcription factor network	75	2 (2.7%)	0.00603	0.0166	PID

Table S6. GO terms of 65 genes with ConsensusPathDB (category level 4) (adjusted *p*<0.01).

Category level	GO term		Set size	Candidates contained	*p*-value	*q*-value
**Biological process**	GO:0034645	cellular macromolecule biosynthetic process	4976	30 (0.6%)	6.54e-06	0.000634
	GO:0009059	macromolecule biosynthetic process	5040	30 (0.6%)	8.62e-06	0.000634
	GO:0009889	regulation of biosynthetic process	4310	27 (0.6%)	1.38e-05	0.000676
	GO:0044271	cellular nitrogen compound biosynthetic process	5019	29 (0.6%)	2.59e-05	0.000881
	GO:0010467	gene expression	5644	31 (0.5%)	2.99e-05	0.000881
	GO:0051171	regulation of nitrogen compound metabolic process	6033	32 (0.5%)	4.06e-05	0.000996
	GO:0080090	regulation of primary metabolic process	6227	32 (0.5%)	8.1e-05	0.0017
	GO:0031323	regulation of cellular metabolic process	6297	32 (0.5%)	0.000103	0.00189
	GO:0060255	regulation of macromolecule metabolic process	6339	32 (0.5%)	0.000119	0.00195
	GO:0018130	heterocycle biosynthetic process	4372	25 (0.6%)	0.000176	0.00259
	GO:0090304	nucleic acid metabolic process	5270	28 (0.5%)	0.0002	0.00267
	GO:1901362	organic cyclic compound biosynthetic process	4529	25 (0.6%)	0.000316	0.00387
	GO:0034654	nucleobase-containing compound biosynthetic process	4307	24 (0.6%)	0.000393	0.00445
	GO:0019438	aromatic compound biosynthetic process	4383	24 (0.5%)	0.000516	0.00542
	GO:0009892	negative regulation of metabolic process	3076	18 (0.6%)	0.00172	0.0169
	GO:0050779	RNA destabilization	36	2 (5.6%)	0.00478	0.0421
	GO:0009893	positive regulation of metabolic process	3654	19 (0.5%)	0.00487	0.0421
	GO:0051101	regulation of DNA binding	124	3 (2.4%)	0.00543	0.0439
	GO:0072175	epithelial tube formation	126	3 (2.4%)	0.00568	0.0439

	GO:0016331	morphogenesis of embryonic epithelium	141	3 (2.1%)	0.00774	0.0569
**Molecular function**	GO:0003677	DNA binding	2515	22 (0.9%)	6.1e-07	7.63e-06
	GO:0001067	regulatory region nucleic acid binding	1535	17 (1.1%)	7.63e-07	7.63e-06
	GO:0046872	metal ion binding	4259	23 (0.5%)	0.000869	0.00579
	GO:0001228	DNA-binding transcription activator activity, RNA polymerase II-specific	444	6 (1.4%)	0.00164	0.00822
	GO:0004879	nuclear receptor activity	52	2 (3.8%)	0.00978	0.0391
**Cellular component**	GO:0031981	nuclear lumen	4461	24 (0.5%)	0.000678	0.0183
	GO:0005634	nucleus	7626	33 (0.4%)	0.00198	0.0183
	GO:0005654	nucleoplasm	4103	21 (0.5%)	0.00341	0.0307

Table S7. Pathway analysis of 65 genes with ConsensusPathDB (adjusted *p*<0.01).

**Pathway name**	**Set size**	**Candidates contained**	***p*-value**	***q*-value**	**Pathway source**
Rheumatoid arthritis - Homo sapiens (human)	93	8 (8.7%)	1.69e-05	0.00776	KEGG
Transferrin endocytosis and recycling	31	4 (12.9%)	0.000533	0.0765	Reactome
Oncogene Induced Senescence	32	4 (12.5%)	0.000603	0.0765	Reactome
Small cell lung cancer - Homo sapiens (human)	92	6 (6.5%)	0.000935	0.0765	KEGG
G1 to S cell cycle control	64	5 (7.8%)	0.00112	0.0765	Wikipathways
Small cell lung cancer	96	6 (6.2%)	0.00117	0.0765	Wikipathways
Adenosine ribonucleotides de novo biosynthesis	38	4 (10.5%)	0.00117	0.0765	HumanCyc
RND1 GTPase cycle	42	4 (9.5%)	0.00171	0.0776	Reactome
Human cytomegalovirus infection - Homo sapiens (human)	225	9 (4.0%)	0.00185	0.0776	KEGG
RND2 GTPase cycle	43	4 (9.3%)	0.00186	0.0776	Reactome
Cyclin D associated events in G1	44	4 (9.1%)	0.00203	0.0776	Reactome
G1 Phase	44	4 (9.1%)	0.00203	0.0776	Reactome
Cyclins and cell cycle regulation	23	3 (13.0%)	0.00269	0.0948	BioCarta
Spinal Cord Injury	117	6 (5.1%)	0.00319	0.104	Wikipathways
Cell cycle: g1/s check point	25	3 (12.0%)	0.00343	0.105	BioCarta
Insulin receptor recycling	26	3 (11.5%)	0.00384	0.109	Reactome
Collecting duct acid secretion - Homo sapiens (human)	27	3 (11.1%)	0.00429	0.109	KEGG
Constitutive Signaling by AKT1 E17K in Cancer	27	3 (11.1%)	0.00429	0.109	Reactome
Lipid and atherosclerosis - Homo sapiens (human)	215	8 (3.7%)	0.00508	0.119	KEGG
Iron uptake and transport	57	4 (7.0%)	0.00523	0.119	Reactome
Superpathway of purine nucleotide salvage	59	4 (6.8%)	0.00591	0.119	HumanCyc
Purine nucleotides de novo biosynthesis	59	4 (6.8%)	0.00591	0.119	HumanCyc
Oxidative phosphorylation - Homo sapiens (human)	133	6 (4.5%)	0.00597	0.119	KEGG
Prion disease - Homo sapiens (human)	273	9 (3.3%)	0.00661	0.126	KEGG
Chemokine receptors bind chemokines	62	4 (6.5%)	0.00704	0.129	Reactome
Vitamin D Receptor Pathway	184	7 (3.8%)	0.00765	0.13	Wikipathways
Viral protein interaction with cytokine and cytokine receptor - Homo sapiens (human)	100	5 (5.0%)	0.00776	0.13	KEGG
Ectoderm Differentiation	142	6 (4.2%)	0.00814	0.13	Wikipathways
Amino acids regulate mTORC1	34	3 (8.8%)	0.00824	0.13	Reactome
ROS and RNS production in phagocytes	35	3 (8.6%)	0.00893	0.135	Reactome
Toll-like receptor signaling pathway - Homo sapiens (human)	104	5 (4.8%)	0.00912	0.135	KEGG
