# Supplementary figures and images for: Effects of Low and High Maternal Protein Intake on Fetal Skeletal Muscle miRNAome in Sheep
Source: Animals (Basel). 2024 May 28;14(11):1594. doi: 10.3390/ani14111594 (PMC11171157; doi:10.3390/ani14111594)

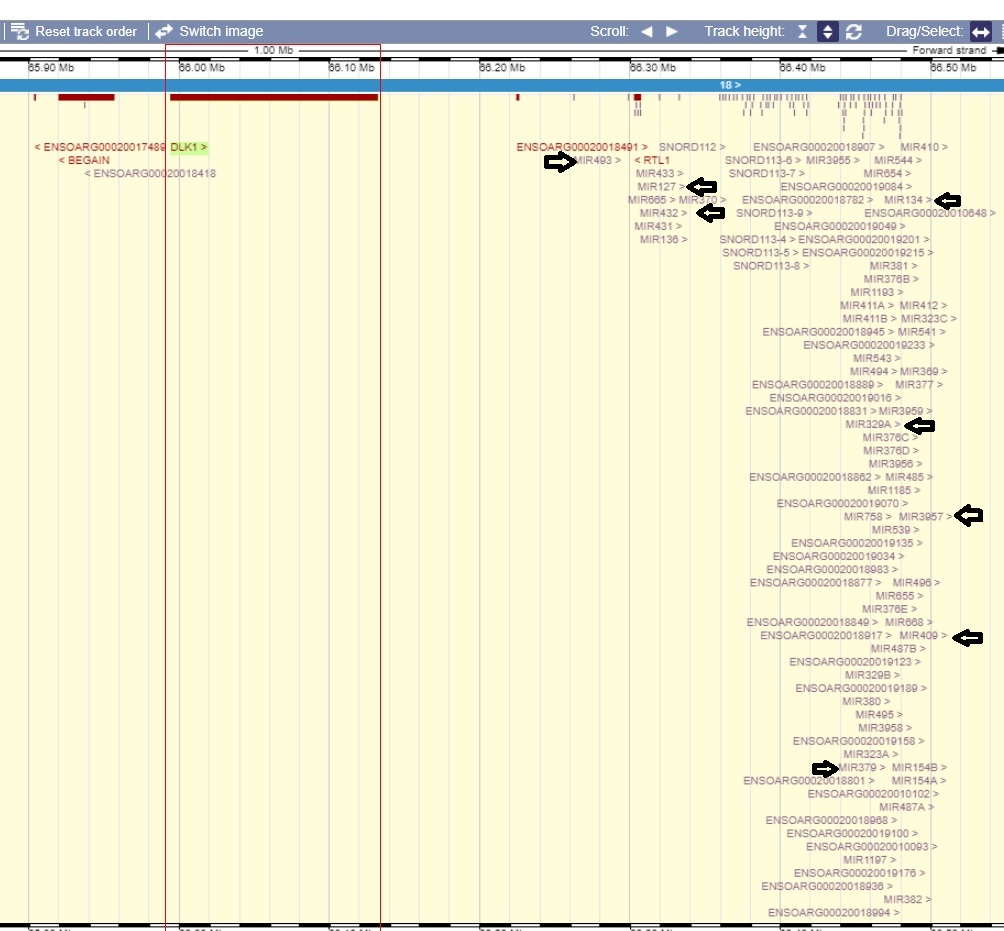

Supplement: Supplementary file 1 [file animals-14-01594-s001.zip › Figure S1.jpg]

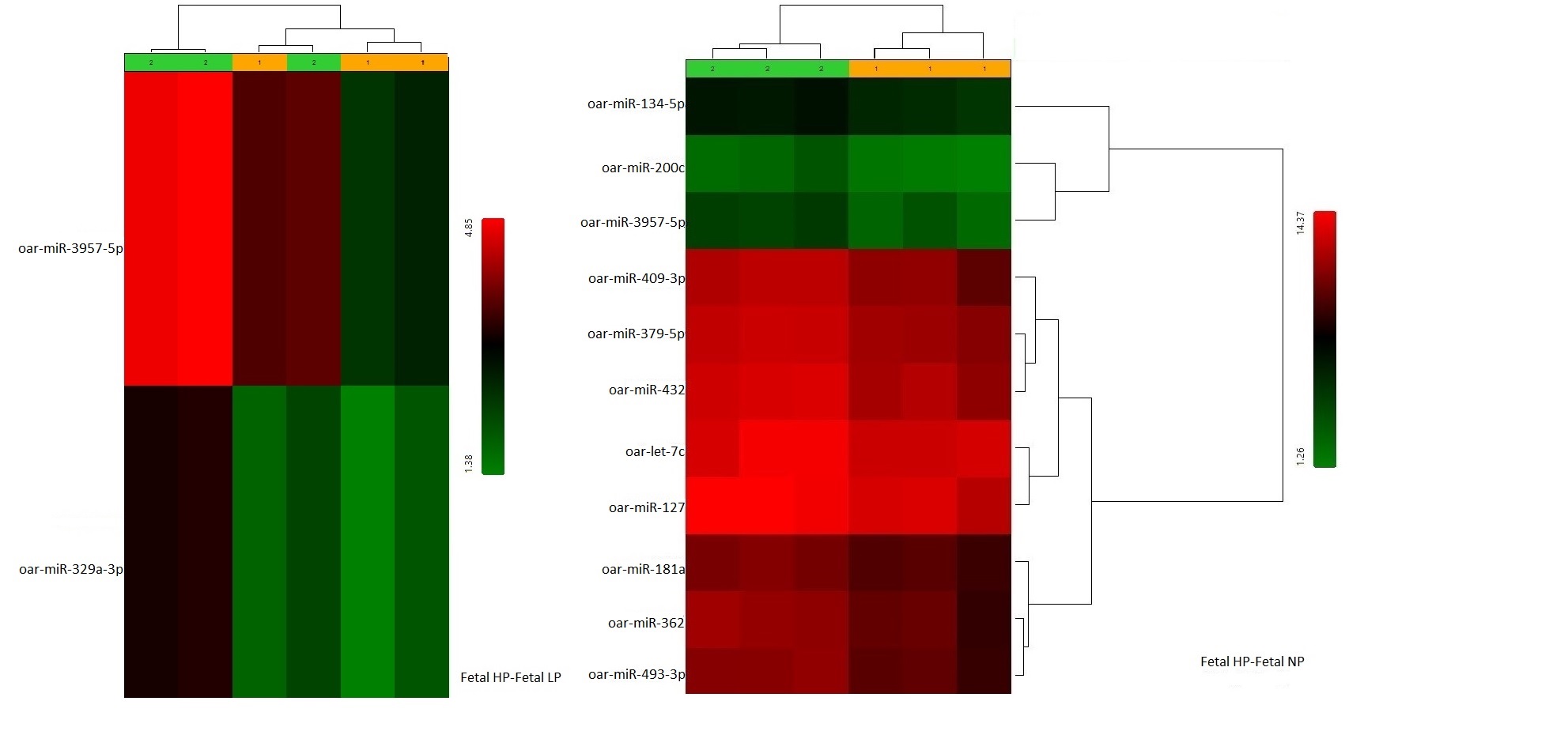

Supplement: Supplementary file 1 [file animals-14-01594-s001.zip › Figure S2.jpg]
